# Experience of adherence to treatment among patients with coronary artery disease during the COVID-19 pandemic: A qualitative study

**DOI:** 10.34172/hpp.2021.59

**Published:** 2021-12-19

**Authors:** Nasrin Zahmatkeshan, Zahra Khademian, Ladan Zarshenas, Mahnaz Rakhshan

**Affiliations:** ^1^Department of Nursing, School of Nursing and Midwifery, Shiraz University of Medical Sciences, Shiraz, Iran; ^2^Department of Nursing, School of Nursing and Midwifery, Community based Psychiatric Care Research Center, Shiraz University of Medical Sciences, Shiraz, Iran

**Keywords:** Coronary artery disease, COVID-19, Qualitative research, Treatment adherence

## Abstract

**Background:** The coronavirus disease 2019 (COVID-19) pandemic has caused patients with chronic diseases to face various challenges. The present qualitative study aimed to explore adherence to treatment in patients with coronary artery disease (CAD) during the COVID-19 pandemic.

**Methods:** This qualitative content analysis was conducted from September 2020 to February 2021. Online in-depth interviews were conducted with 15 patients with CAD after discharge from Nemazi and Al-Zahra heart hospitals, Shiraz, Iran. Data management was done via MAXQDA 12 software using conventional content analysis based on the method proposed by Graneheim and Lundman.

**Results:** The results revealed three main categories, nine subcategories, and 431 primary codes. The first category was ‘improved self-care in the shadow of COVID-19‘ (Improving self-care due to fear of COVID-19, ‘utilization of alternative strategies, and reinforcement of self-care beliefs). The second category was ‘redefinition of support systems’ (need for a support system, seeking for alternative support systems, and changes in social interactions). The last category was ‘barriers to treatment adherence’ (shortage of financial resources, need to adjust with working conditions, and mental conflicts).

**Conclusion:** The results indicated that the COVID-19 threats encouraged the patients with CAD to adhere to their care principles. Nonetheless, the restrictions resulting from the pandemic caused problems in adherence to treatment. Thus, redefinition of the support systems in accordance with the present conditions are recommended.

## Introduction

The emergence of coronavirus disease 2019 (COVID-19) has turned into an unprecedented challenge all over the world. This disease quickly changed into a pandemic, exerting a huge effect on public health as well as on social and economic activities.^1^ In this context, the exponential growth in the number of patients with COVID-19 during the past year affected healthcare systems in a large number of countries all around the world. Since no definite and effective treatment has been found for this disease up to now, more than 140 million people have been infected and more than three million deaths have been reported during the past year.^2^ In Iran, more than 2 200 000 people have been infected and more than 66 000 deaths have been reported until now (April 2021).^3^

Evidence has shown the highest risk of COVID-19 infection among the elderly individuals with underlying disorders like diabetes, immune system deficiency, and coronary artery diseases (CADs).^4^ Moreover, patients with a long history of CAD and those with the risk factors of atherosclerotic cardiovascular disease have been found to be at a higher risk of acute coronary conditions during acute COVID-19 infection.^5^ Additionally, a study in China showed 51.2% mortality rate among the patients with heart attack who were infected with COVID-19.^6^ In another study in Iran, the mortality rate of COVID-19 was 27.8% among the patients who suffered from CAD.^7^

Generally, the preventive measures taken for preventing the further prevalence of COVID-19 can indirectly affect the outcomes among patients with chronic diseases, particularly those suffering from CAD. For instance, traffic restrictions and quarantine as well as increased probability of pollution in treatment settings and hospitals can affect the accessibility of care services for patients with chronic diseases.^8^ Previous studies have indicated that patients with chronic diseases delayed their referral for receiving healthcare services during the COVID-19 pandemic.^9,10^ Moreover, these patients’ access to community-based care services was accompanied by challenges, which could enhance the indirect effects of COVID-19.^11^ This could also result in unwanted complications in such diseases as CAD that are accompanied by increased complications and fast progress.^12^

In general, adherence to treatment in CAD ranges from 40%-60%.^13^ This measure has been reported to be 59% in Iran.^14^ On the other hand, chronic diseases can be weakly managed in developing countries due to shortage of health resource.^15^ Therefore, a serious situation can be predicted among people in developing countries.^8^ Yet, appropriate health outcomes can be achieved by adherence to recommended treatments among patients with CAD, which is of particular importance.^16,17^ However, the challenges occurring during the pandemic may affect the patients’ adherence to treatment and ruin the health achievements obtained prior to this condition.^8^ Thus, this issue has to be explored during the COVID-19 pandemic.

Given that COVID-19 is a newly emerged disease, scarce quantitative and qualitative information is available regarding its possible impacts on the outcomes of patients suffering from CAD. Up to now, several studies have been conducted on adherence to treatment among patients with chronic diseases during the COVID-19 pandemic, and some have focused on adherence to medications during the pandemic.^18,19^ Another research evaluated the barriers against self-care among patients with type II diabetes during lockdown.^20^ However, less attention has been paid to the experience of adherence to treatment among patients with chronic diseases, specifically those with CAD, as a high-risk group. Considering the importance of these cares in improving the health outcomes of patients with CAD, investigation of their experiences of adherence to treatment during the pandemic can provide novel perspectives and approaches for designing interventions and making policies. Regarding the importance of understanding the patients’ experiences of the effects of the disease on their treatment processes in various cultural contexts, a qualitative method seems to be appropriate for studying this phenomenon. This research method can determine the patients’ experiences of the effects of the pandemic on their adherence to treatment. Hence, the present qualitative study aimed to explore adherence to treatment among patients with CAD during the COVID-19 pandemic.

## Materials and Methods

This qualitative content analysis was performed using conventional content analysis on 15 patients with the history of admission in cardiac intensive care units in Nemazi and Al-Zahra heart hospitals, Shiraz, Iran, in the last six months. Participants were selected purposefully based on the inclusion criteria which included definite diagnosis of CAD, age above 18 years, stable cardiovascular conditions, and willingness to take part in the research. Data collection was continued until data saturation. In this regard, no new data were obtained from the 13^th^ to the 15^th^ interviews, and the previous data were repeated.

The data were collected through individual semi-structured in-depth interviews conducted via voice and video calls, which were made when the participants’ convenience and in their homes. One of the researchers (the first author) who was a PhD candidate in nursing and was experienced in taking care of cardiac patients conducted the interviews.

The interviews started with general questions, such as “how has COVID-19 affected your adherence to treatment for controlling your CAD” and “what challenges have you faced as to self-care during the COVID-19 pandemic”. Based on the research objectives, these questions aimed at guiding the interviews and had open answers. Thus, the participants’ responses directed the interviews towards achieving the main research objective. Then, the interviews were continued using probing questions, such as “can you give an example” and “can you explain more”, in order to perceive the participants’ experiences of adherence to treatment. The interviews averagely ranged from 30 to 60 minutes. It should also be mentioned that the study objectives were explained to the patients. After obtaining the participants’ consent, the interviews were recorded by a cellphone. The analysis was performed simultaneously with data collection in a constant comparative manner. Data management was done using the MAXQDA 12 software, and data analysis was done using the method proposed by Graneheim and Lundman.^21^ In doing so, the interviews were transcribed immediately after they were finished. Then, the whole transcript was read to gain an overall understanding of the interview content, meaning units were determined, and primary codes were extracted. After that, similar codes were merged to form subcategories, and subcategories together formed the main categories.

In this study, the trustworthiness of the data was assessed using the method proposed by Lincoln and Guba.^22^ Accordingly, data credibility was enhanced via prolonged engagement throughout the study and the data collection process. In addition, the researcher had the experience of working with patients suffering from CAD. Besides, no predefined frameworks were taken into account for data collection and analysis. Moreover, the results were returned to the participants in order to determine the accuracy, completeness, and conformity of the interpretations to their experiences and to ensure the accuracy of the codes. At this stage, the codes that did not transfer the participants’ experiences were modified. Furthermore, for data dependability, some samples of interview transcripts, together with the related codes, concepts, and categories, were assessed by experts in qualitative studies. Besides, audit trail was used to achieve the reliability, neutrality, and objectivity of the data. In doing so, all research information was retained so that it could be audited by other researchers. For data confirmability, all research data were properly stored to facilitate the future checking and validation of results. Finally, transferability was determined by complete introduction of the research proposal and application of maximum variation in selecting the participants.

## Results

In this study, interviews were conducted with 15 patients (six females and nine males) with CAD. The mean age of the participants was 62.4 ± 5.5 years (ranging from 48 to 72 years). In addition, five, six, two, and two participants had primary school, middle school, diploma, and academic degrees, respectively (Table 1).

**Table 1:** Demographic characteristics of the study participants

**Participant**	**Age (y)**	**Gender**	**Marital status**	**Educational level**	**Occupation**
Participant1	66	Male	Married	Primary school	Free job
Participant 2	67	Male	Married	Primary school	Free job
Participant 3	55	Female	Married	Diploma	Housekeeper
Participant 4	66	Male	Married	Middle school	Free job
Participant 5	63	Female	Widow	Middle school	Housekeeper
Participant 6	64	Female	Widow	Primary School	Housekeeper
Participant 7	58	Male	Married	Middle school	Free job
Participant 8	64	Female	Married	Middle school	Housekeeper
Participant 9	60	Male	Married	Middle school	Retired
Participant 10	61	Female	Married	Primary school	Housekeeper
Participant 11	58	Male	Married	Diploma	Free Job
Participant 12	72	Male	Married	Primary school	Free job
Participant 13	48	Male	Married	Academic education	Employer
Participant 14	49	Male	Married	Academic education	Employer
Participant 15	60	Female	Married	Middle school	Housekeeper

Data analysis revealed three main categories, nine subcategories, and 431 primary codes. The main categories were ‘improved self-care in the shadow of COVID-19 threats’, ‘redefinition of support systems’, and ‘frustration due to helplessness in treatment’ (Table 2).

**Table 2:** Adherence to treatment categories and subcategories among patients with coronary artery disease during the COVID-19 pandemic

**Category**	**Subcategory**
Improved self-care in the shadow of COVID-19	Improving self-care due to fear of COVID-19
	Utilization of alternative strategies
	Reinforcement of self-care beliefs
Redefinition of support systems	Need for a support system
	Seeking for alternative support systems
	Changes in social interactions
Barriers to treatment adherence	Shortage of financial resources
	Need to adjust with working conditions
	Mental conflicts

### Improved self-care in the shadow of COVID-19 

The threats imposed by COVID-19 on patients with CAD and their conditions were mentioned as one of the important factors in improving the level of self-care. This category consisted of three subcategories, namely ‘improving self-care due to fear of COVID-19’, ‘utilization of alternative strategies’, and ‘empowerment of self-care beliefs’.

*Improving self-care due to fear of COVID-19*

According to the participants, fear from the effects of COVID-19 on the cardiac disease process could foster the application of the treatment advice. In this regard, one of the participants talked about his attempts for application of the treatment recommendations:

*“It has been claimed that COVID-19 is dangerous for patients with heart diseases and affects them more. I try to be careful. I wasn’t careful in the past, but now I carefully adhere to whatever my doctor says. I have my medications on time without any interruptions”* (participant 4, male, 66 years old).

Another participant talked about her attention to treatment advice due to suffering from two underlying diseases:

*“I have diabetes and previously I had a MI. It has been expressed that these two diseases are more affected by the Corona virus, resulting in higher mortality rates. I’m very careful. I check my blood sugar level using a device every day to ensure that it has not increased. I’m also careful about what I eat; I am on diet food. Besides, I take my medicines on time”* (participant 6, female, 64 years old).

*Utilization of alternative strategies*

Due to the limitations during the COVID-19 pandemic, the majority of the participants faced numerous challenges in self-care. Therefore, in order to improve their compatibility with these conditions, some patients were creatively seeking for new methods to overcome their problems and adhere to the recommended treatments. In this context, one of the participants said:

*“Since the pandemic has started, I’ve not been able to go out; I go to the yard and walk around the yard for nearly 20 minutes and breathe deeply…”* (participant 8, female, 64 years old).

Considering adherence to medication regimen, one of the participants stated:

*“I take my medicines before I go out in the morning, but I forget in the afternoon. I adjust my cellphone alarm to ring, so that I won’t forget”* (participant 4, male, 66 years old).

Another participant maintained:

*“These days, bad news causes me to feel worse. When I see something nerve-racking, I sit at a quiet place and practice the relaxation techniques I learned in classes to get calm”* (participant 2, male, 67 years old).

*Reinforcement of self-care beliefs*

Several participants stated that they made genuine attempts to enhance their care power during the pandemic by relying on their capabilities in order to control the high risk of being infected with the virus. According to the majority of the participants, further care was required during the pandemic. They believed that they could prevent further complications by controlling the signs and symptoms and adhering to the care advice. In this regard, one of the participants said:

*“I’ve always been firm; I have passed more difficult situations. When I had a heart attack, the doctor told me that it was a serious attack, but I was careful, and the heart problem was controlled. Now, nothing has happened…; a disease has emerged, I know how to take care of myself. It’s right, they say that heart patients are at a higher risk, but if people do what they should, nothing will happen”*(participant 1, male, 66 years old).

Another participant maintained:

*“I believe that instead of being afraid and telling myself that I have a heart problem, I may be infected with the virus, and I may die, I should be careful and take my medicines on time. If I wasn’t careful in the past, I should be careful now. I should adhere to what doctors and nurses say until the disease is gone; it will not remain for good”* (participant 7, male, 58 years old).

Another participant emphasized the importance of paying attention to the health status during the pandemic:

*“Nothing is more important than staying healthy in this situation. My heart problem is under control. I know this improvement is due to following the advice given to me. I am still taking care of myself. I know that I will not have any problems here either* (participant 11, male, 58 years old).

### Redefinition of support systems

This category consisted of three subcategories, including ‘need for a support system’, ‘seeking for alternative support systems’, and ‘changes in familial and social interactions’. The study results revealed the important role of support in adherence to treatment among the patients with CAD during the pandemic. These supports were effective in strengthening the adherence to care advice.

*Need for a support system*

According to the participants, receiving support from their acquaintances increased their self-confidence and coping with stress, eventually improving their health status. Given the limitations of CAD during the COVID-19 pandemic, the received supports played a critical role in their adherence to treatment. In this context, support on the part of the patient’s spouse and children was of utmost importance. One of the participants maintained:

*“During the pandemic, my wife played a key role in my adherence to the treatment plans; she planned for visiting the doctor, she reminded me to take my medicines, and she called me and told me to be careful when I was at work. She was also careful about my diet and other things. I owe my life to her. She has really devoted her time to me”* (participant 13, male, 48 years old).

Another study participant stated:

*“My husband is always worried about my health status. He doesn’t let me go out for shopping or other works. He prepares everything I need. Now that I’m home all the time, he is careful so that I’m not depressed or bored…Sometimes, he takes me to less crowded areas in the suburbs and we take a walk”* (participant 8, female, 64 years old).

*Seeking for alternative support systems*

The majority of the study participants stated that due to limitations for presence in public and private clinics during the pandemic, they had lower access to physicians and nurses. They faced problems in the continuation of their treatment programs. Therefore, they made use of indirect systems to gain information for eliminating their problems. In this regard, one of the participants said:

*“In the last visit, the doctor changed my medicines. After I consumed the new medications, I felt weak. I didn’t know whether it was due to the new medicines or something else…I was afraid to go to clinics, because they were crowded. I didn’t know whether to take or not to take the medicines. Finally, I called the telephone consultation center and asked for consultation”* (participant 9, male, 60 years old).

Another participant maintained:

*“In the past, I used to go to the doctor whenever I had a problem. Sometimes, I even went earlier than my appointment. However, I was afraid during the pandemic…I sometimes call my nurse or watch TV programs about the Corona virus, so that I can care for myself more efficiently”* (participant 2, male, 67 years old).

*Changes in social interactions*

Based on the study participants, the communication restriction associated with COVID-19 affected their social interactions with their families and friends. In this respect, one of the participants stated:

*“In the past, my children used to pay me a visit every day…but they go to work and are afraid of being infected with the virus and transmitting the disease to me. I don’t insist. I miss them, but I say it doesn’t matter, I’m not weak, and I can take care of myself. I tell them not to be worried about me”* (participant 6, female, 64 years old).

In some cases, the restrictions reduced the patients’ dependence and increased their self-care capabilities. In this regard, one of the participants said:

*“My children are not here. My sister mostly helps me. Due to the pandemic, I told her not to come here and she did so…I’m really dependent on her, but I try to do my works and not to disturb her”* (participant 5, female, 63 years old).

The restrictions also made the patients aware of their capabilities. For instance, one of the participants maintained:

*“When my children used to carry out my duties, I felt that I was weak and needed someone to take care of me. Now that they visit me less often, I see I can do my works and I don’t have any problems”* (participant 4, male, 66 years old).

*Barriers to treatment adherence*

This category consisted of three subcategories, i.e. ‘disruption in treatment programs due to financial restrictions during the pandemic’, ‘need for readjustment of self-care based on occupational conditions’, and ‘weakness in follow-up of treatment due to mental conflicts’. The majority of the participants stated that although they made genuine attempts to follow up their treatments during the COVID-19 pandemic, the uncontrollable conditions sometimes resulted in their frustration, affecting their adherence to treatments.

*Shortage in financial resource*

Due to the direct and indirect effects of the COVID-19 outcomes on economic conditions, most of the participants were affected by the pandemic. In this context, inappropriateness of the prescribed diets to financial power, lack of financial resources for preparation of protective equipment, high treatment expenditures, and high cost of medications were mentioned as barriers against complete adherence to the treatment program. One of the participants stated:

*“I had to hire a worker for my shop, so that I didn’t have to go to work and have contact with people, but the worker does not work as I do…My income has reduced to half. Now consider the costs of life, medications, treatment, diet food,…How can I adhere to everything I have been told”* (participant 11, male, 58 years old).

Another participant mentioned lack of medicines and their high costs as a factor in non-adherence to consumption of medications:

*“Plavix is not in the market. My doctor has told me to take this medicine on a regular basis. It was finished, and I wasn’t able to buy the medicine anymore. After two weeks, one of my son’s friends found two packs, but three times more expensive than its actual cost”* (participant 10, female, 61 years old).

Reduction of support services and insurance supports was yet another factor in non-adherence to treatments. In this respect, one of the participants said:

*“The government has to do something for patients like me. Echocardiography costs 400-500 thousand Tomans. How much should we pay for medications? The insurance only covers one-fourth of the costs. How can someone like me adhere to the advice during the recent recession? I don’t go to the doctor, I don’t undergo echocardiography, and I don’t take medicines even if I have to”* (participant 12, male, 72 years old).

*Need to adjust with working conditions*

One of the challenging situations in adherence to treatment during the COVID-19 pandemic was related to work and workplace restrictions. Need for being active, working, and earning a living on one hand, and the necessity to avoid referring to crowded places on the other hand caused a challenge for the patients, in such a way that they were obligated to make changes in their work conditions. In this regard, one of the participants maintained:

*“I have lower income during the pandemic. I have to work harder. Sometimes, fatigue causes me to forget to take my medications. I go out early in the morning until late at night. My medicines are in the gloves compartment, but I realize that I have forgotten to take them when I see them…I try to work less…but sometimes nothing can be done”* (participant 2, male, 67 years old).

Considering the problems which resulted from the restrictions, another participant mentioned:

*“I have to wear two masks when I’m at work. I wear them since morning when I go out until night…I have shortness of breath, my heart beats faster, I feel I’m choking…Even when I remove the masks, lines remain on my face for a long time”*(participant 7, male, 58 years old).

Some participants tried to adjust themselves with these restrictions. They did their daily activities and, at the same time, followed up their care and preventive programs. One of the participants stated:

*“I have to go to work, I’m stressed out, my heart beats fast…I changed my workplace, I went to a place where I had less contact with people…I feel calm now…I try to be careful about myself”* (participant 13, male, 49 years old).

*Mental conflicts*

Generally, the pandemic led to different mental conflicts among the patients due to fear from being infected with the disease or transmitting the disease to others, stress and anxiety resulting from listening to bad news, and feeling unable to control the conditions, which caused problems and hopelessness in continuation of their treatment programs. In addition, the unknown nature and continuity of social restrictions caused disruptions in self-care programs in some participants, in such a way that they felt hopeless for continuing their treatment programs. As an instance, one of the participants maintained:

*“I’m tired; everyone who sees me tells me to be careful and not to go out of the house…I don’t know when this will finish. I can’t be careful about my blood pressure, eat diet food, and take my medicines on time every day…I sometimes say I have to let it go, I may die in the end”* (participant 9, male, 60 years old).

Another participant said:

*“Our neighbor who had COVID-19 died a few days ago… he had hypertension. I was so afraid that I wasn’t able to sleep at night, I had a tight feeling in my chest…I have been very careful, but I’m afraid of being infected and dying at the end”* (participant 2, male, 67 years old).

Another participant stated:

*“I feel depressed because of staying home all the time. I don’t like to adhere to any advice anymore”* (participant 15, female, 60 years old).

## Discussion

This study aimed to explore the experience of adherence to treatment among the patients with CAD during the COVID-19 pandemic. Based on the findings, the participants’ experiences were divided into three categories, namely ‘purposeful self-care in the shadow of COVID-19 threats’, ‘redefinition of support systems’, and ‘frustration due to helplessness in treatment’. These results represented the opportunities and challenges regarding adherence to treatment during the pandemic.

The unknown nature of COVID-19, as well as lack of a definite and effective treatment, caused the patients with CAD to be fearful and worried about their health status. Evidence has indicated that the incidence of epidemics has always been accompanied by such outcomes as panic.^23,24^ For instance, one-third of the participants in a survey in China reported moderate to severe fear from COVID-19.^25^ In addition, a higher level of fear has been detected among individuals suffering from chronic diseases.^26^ Fear has been defined as a response, which can affect people both positively and negatively.^27^ It seems that the initial fear and anxiety experienced by the patients with CAD provided a great opportunity for them to create motivation and change their behaviors. In fact, these patients increased their cardiac care levels and followed the treatment recommendations more carefully. Other studies conducted on chronic diseases have also shown fear as a stimulant for enhancing self-care advice.^27-29^ In the present study, for better adherence to treatment, some participants utilized alternative strategies in order to improve their purposeful self-care during the pandemic. For instance, they tried to be creative in their diets, exercise at home, use the mass media to increase their care ability, and use Internet applications and new technologies to communicate with their friends. Evidence has indicated that patients with chronic diseases used shortcuts or bypassed restrictions for more efficient adherence to treatment.^4^ Besides, new electronic technologies, online shopping applications, and social media^18^ have improved the patients’ access to better care services.

Empowerment of self-care beliefs was another factor in promotion of purposeful care. Generally, patients with CAD are considered as the high-risk group, because of a higher risk of infection and mortality among them. The majority of the current study participants stated that having a positive attitude towards the care process, focusing on health status, and putting emphasis on adherence to treatment advice were effective in improvement of self-care during the pandemic. A prior qualitative study conducted on patients with diabetes also demonstrated that the participants regarded themselves as a high-risk group for COVID-19 infection and had to care for themselves efficiently. On the other hand, they believed that they had to change their attitudes about their vulnerability, follow their treatment process by adhering to the recommended measures, and develop a positive attitude towards the treatment process and controlling the disease conditions.^30^

In the present study, the participants’ support systems were found to be effective in their adherence to treatment during the COVID-19 pandemic. The majority of the participants mentioned that their families played a pivotal role in their adherence to treatment during the pandemic. Accordingly, they felt calm in the shadow of their families’ support and made attempts towards self-care. The supportive role of families during the pandemic has been expressed in other studies, as well.^18,31,32^ In fact, family members have been introduced as the most accessible and reliable sources of mental support^28,33^; their absence could disrupt the patients’ daily cares.^20^ However, due to the existing restrictions and need for further support during the COVID-19 pandemic, the patients’ social interactions were affected, eventually directing them towards promotion of their capabilities. In other words, necessity for self-care encouraged the patients to rely on their capabilities for meeting their needs, thereby causing them to feel independent. A similar study revealed a significant, direct relationship between self-care and self-efficacy among patients with cardiac diseases.^34^ Nonetheless, due to such measures as quarantine and social restrictions taken for preventing the further prevalence of COVID-19, the patients with CAD faced challenges for having access to their medical and treatment services. In addition, they were confused in the process of continuation of their treatment, because of lack of access to physicians and nurses. Moreover, increased probability of being infected with the virus in hospitals and treatment settings deprived them from receiving medical consultations and health services. Therefore, the restrictions directed the patients towards indirect treatment supports. The majority of the participants gained the necessary information from telephone-based service systems, the virtual space, and the media. Also, in a study performed in England, 67% of healthcare providers reported that access to healthcare services was hard for patients during the COVID-19 pandemic, which was effective in their adherence to treatment.^35^ However, supervising institutions have suggested that patients with chronic disorders should not stop referring to healthcare centers and disrupt their treatments due to fear from the consequences of the pandemic.^36^ In such periods, telephone contacts, text messages, social media, and delivery services for delivering medications to patients’ houses have been recommended for better adherence to treatment.^18^

Another category which emerged in the present study was frustration due to helplessness in treatment. In spite of the patients’ attempts to apply the treatment advice during the pandemic, the uncontrollable conditions sometimes affected the treatment programs, so that the patients sometimes discontinued their treatments. Moreover, lack of financial resources for purchase of medications and protective equipment, follow-up, and adherence to diets caused challenges for the patients’ adherence to their care programs. Conflicts between presence in the workplace and adherence to treatment programs also caused problems for the patients. Evidence has indicated that periodic occupations were at the highest risk during the COVID-19 pandemic, and individuals with chronic diseases were at a high risk since they had to be present at their workplace to earn a higher wage for meeting the costs of living and care.^37^ High costs of living and treatment as well as inability to follow up treatment were also among the patients’ concerns.^38^ Limited financial resources affected the patients’ adherence to medications, as well. Shortage or high cost of medications affected the patients’ access to their required medicines, so that they had to reduce or discontinue their medications intake. Other studies have also shown the reduction or discontinuation of medications consumption due to fear from their side effects among patients with chronic diseases.^28,39^ Previous studies have also revealed the reduction of the dose or discontinuation of medications due to access restrictions, which was followed by increase in disease complications^19,40^ and occupational issues.^41^

In addition to what was mentioned above, fatigue and hopelessness resulting from the uncontrollable conditions, anxiety due to listening to bad news, and unpleasant outcomes in similar patients caused the patients to suffer from helplessness. Similarly, previous studies revealed stress and anxiety as common reactions during critical situations.^42,43^ The negative impacts of COVID-19 prevalence could further affect the clinical outcomes among patients with chronic diseases, such as CAD, which could be intensified by stress and anxiety.^44^ In a research carried out in England, 80% of healthcare workers reported a worse mental health status among patients with chronic diseases during the COVID-19 pandemic.^35^ Social isolation and the resultant limitations were effective in the lives of most patients with chronic diseases, as well. In this regard, Hu et al disclosed that social isolation exposed the elderly people to a higher risk of negative emotions, such as depression and anxiety,^45^ eventually reducing adherence to treatment and self-management.^20^

The present study was among the first investigations on the experiences of adherence to treatment among the patients with CAD who are regarded as a high-risk group in the COVID-19 pandemic. The qualitative method used for exploration of the patients’ experiences in the Iranian culture provided a rich description of their challenges for adherence to treatment during the pandemic. However, the research limitation was that the data were collected through online interviews because of the outbreak of COVID-19, while telephone-based interviews have been criticized due to difficulties in establishing relationships and responding to visual cues.

## Conclusion

The study findings demonstrated that the patients with CAD coped with the COVID-19 threats by promoting their purposeful self-care and attempted to adhere to treatments more efficiently. In this context, they were obligated to redefine their support systems. Nonetheless, they sometimes encountered obstacles on their way and felt frustrated. An important finding was the achievement of independence and awareness of their capabilities in some patients due to limited access to home caregivers. Hence, correct perception of these challenges and opportunities can help develop the body of nursing knowledge and help the nurses and other healthcare providers improve the quality of care for these patients, particularly during pandemics. Moreover, considering the nature and diversity of these perceived barriers and opportunities, designing care programs can be effective in facilitation of adherence to the recommended treatments among patients with CAD during pandemics. It is recommended that future studies design programs based on the current findings to improve treatment adherence in patients with CAD during COVID-19 pandemic and evaluate its effectiveness.

## Acknowledgments

The present manuscript was extracted from the Ph.D thesis by Ms. N. Zahmatkeshan. The authors would like to thank the Vice-Chancellor for Research Affairs of Shiraz University of Medical Sciences (Shiraz, Iran) for the financial support. We would also like to express our gratitude to the managers and personnel of the teaching hospitals and all study participants. The authors would also like to thank Shiraz University of Medical Sciences, Shiraz, Iran and also Center for Development of Clinical Research of Nemazee Hospital and Dr. Nasrin Shokrpour for editorial assistance.

## Funding

This study was financially supported by the Vice-Chancellor of Research of Shiraz University of Medical Sciences (project no 17508-08-01-97). The funding body did not participate or play any role in the study design, data collection, data analysis, or reporting the results.

## Competing interests

The authors declare that there is no conflict of interest.

## Ethical approval

This study was approved by the Ethics Committee of Shiraz University of Medical Sciences (code: IR.SUMS.REC.1398.575). At the beginning, the participants were provided with complete information about the study objectives and procedures as well as their freedom for cooperation or withdrawal from the research. They were also assured about the confidentiality of their information. Then, written informed consent forms for taking part in the research and recording the interviews were obtained from the participants. The time of the interviews was also arranged with the participants, so that they had sufficient time for participation in the interviews. It should also be noted that the participants were paid for their Internet phone calls.

## Authors’ contributions

All authors made substantial contributions to the concept and design of the study. NZ collected the data. NZ and ZKh conducted data analysis and interpretation. MR and LZ also participated in data interpretation. NZ and ZKh drafted the manuscript. All authors revised the manuscript critically and finally approved the manuscript. All authors believe that the manuscript reflect meticulous research work.

## Disclaimer

The authors claim that no part of this paper is copied from other sources.
